# Making mouse transcriptomics deconvolution accessible with immunedeconv

**DOI:** 10.1093/bioadv/vbae032

**Published:** 2024-02-28

**Authors:** Lorenzo Merotto, Gregor Sturm, Alexander Dietrich, Markus List, Francesca Finotello

**Affiliations:** Department of Molecular Biology, Digital Science Center (DiSC), University of Innsbruck, Innsbruck 6020, Austria; Biocenter, Institute of Bioinformatics, Medical University of Innsbruck, Innsbruck 6020, Austria; Boehringer Ingelheim International Pharma GmbH & Co KG, Biberach 88400, Germany; Data Science in Systems Biology, TUM School of Life Sciences, Technical University of Munich, Freising 85354, Germany; Data Science in Systems Biology, TUM School of Life Sciences, Technical University of Munich, Freising 85354, Germany; Department of Molecular Biology, Digital Science Center (DiSC), University of Innsbruck, Innsbruck 6020, Austria

## Abstract

**Summary:**

Transcriptome deconvolution has emerged as a reliable technique to estimate cell-type abundances from bulk RNA sequencing data. Unlike their human equivalents, methods to quantify the cellular composition of complex tissues from murine transcriptomics are sparse and sometimes not easy to use. We extended the immunedeconv R package to facilitate the deconvolution of mouse transcriptomics, enabling the quantification of murine immune-cell types using 13 different methods. Through immunedeconv, we further offer the possibility of tweaking cell signatures used by deconvolution methods, providing custom annotations tailored for specific cell types and tissues. These developments strongly facilitate the study of the immune-cell composition of mouse models and further open new avenues in the investigation of the cellular composition of other tissues and organisms.

**Availability and implementation:**

The R package and the documentation are available at https://github.com/omnideconv/immunedeconv.

## 1 Introduction

Bulk RNA sequencing (RNA-seq) captures the expression profiles of complex tissues, but this description averages the transcriptional variability across individual cells. Characterizing the cellular composition of tissues is essential; not only can it affect the differential analysis of gene expression, but its evolution in space and time provides insights on physiological changes in health and disease, e.g. in response to a disease affecting specific cell types. To characterize the cellular composition of complex tissues from transcriptomic data, several computational techniques have been developed. These methods use either cell-type-specific gene sets or expression signatures to compute abundance scores or cell fractions via deconvolution of RNA-seq data, respectively (Finotello and Trajanoski 2018). immunedeconv (Sturm *et al.* 2019) is an R package that offers simplified access to several methods focused on the quantification of immune cell types, unifying input and output data types, and providing a common nomenclature for the quantified cell types. Since its release, immunedeconv has become a popular tool for immuno-oncology thanks to its unique capability to provide easy access to six tools for the quantification of human immune cell types. Here, we present an extended version of immunedeconv which also allows the deconvolution of mouse transcriptomics (https://github.com/omnideconv/immunedeconv).

## 2 Extension of immunedeconv to the analysis of mouse transcriptomics

**Figure 1.**
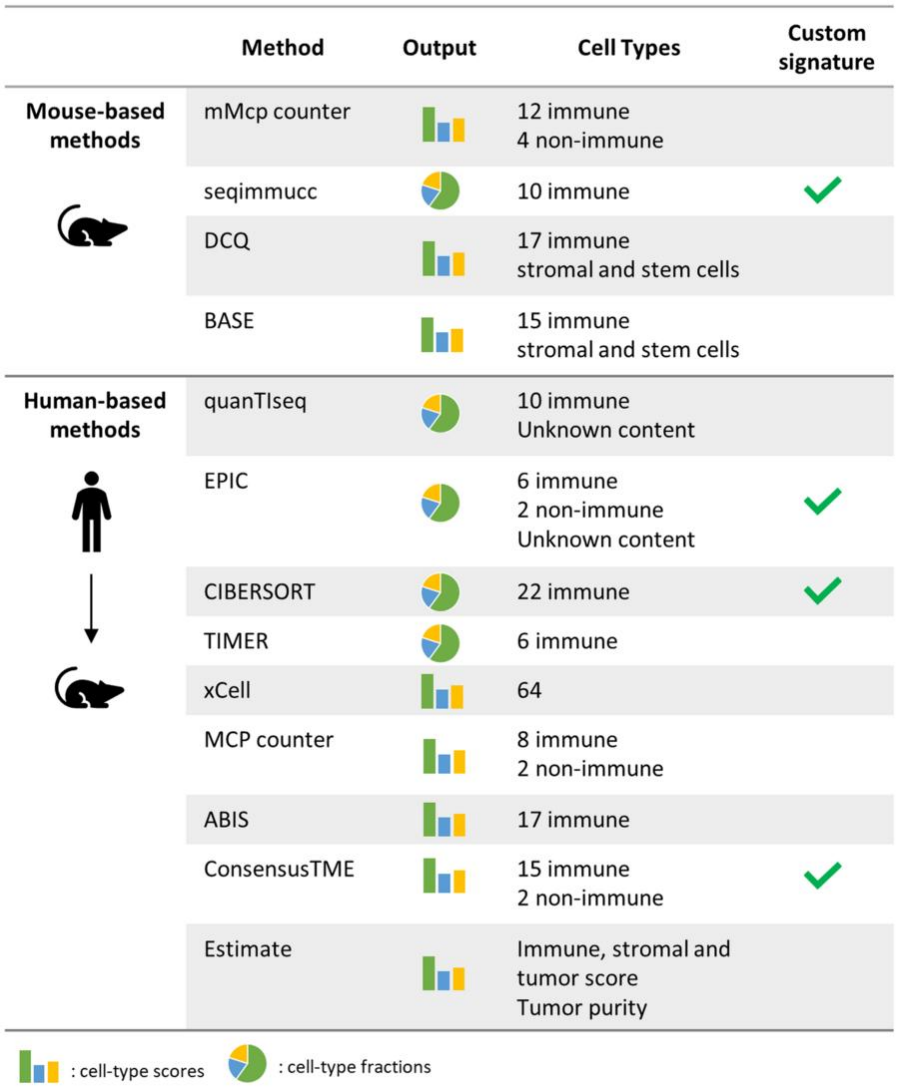
Overview of the methods included in immunedeconv that can be applied to mouse transcriptomics data.

We extended the immunedeconv R package to include four methods to estimate the immune-cell composition of murine samples profiled with RNA-seq data (Fig. 1 and Supplementary Table S1). Murine Microenvironment Cell Population Counter (mMCP-counter) (Petitprez *et al.* 2020) is a method to derive abundance scores for 14 immune and stromal cell types, computed considering the median expression of a set of manually-curated marker genes. seqImmuCC (Chen *et al.* 2018) is a deconvolution method that infers cell fractions for ten immune cell types using a signature matrix derived from mouse RNA-seq data coupled with either support vector regression or least squares regression. Digital cell quantification (DCQ) (Altboum *et al.* 2014) is another regression-based deconvolution method, introduced to investigate immune-cell population changes in mouse samples during infection. It provides a signature matrix with more than 200 immune-cell phenotypes, which allows for an investigation at fine-grained resolution. Binding Association with Sorted Expression (BASE) (Varn *et al.* 2016) is a gene-set-based approach computing abundance scores for more than 180 immune-cell phenotypes. For both DCQ and BASE, the scores can be grouped to obtain estimates for 19 major immune cell types. Prior to their integration into immunedeconv, most of these methods were hard to use due to the unavailability of user-friendly, dedicated packages and/or lack of curated documentation.

In addition, we extended the applicability of human-based deconvolution methods to murine data by implementing a module that converts murine genes in the input bulk RNA-seq data to human ones through orthologous gene mapping (details in Supplementary Methods). Thanks to this module, any human-based method supported by immunedeconv can be used to deconvolve mouse transcriptomics. Six methods were originally implemented in immunedeconv: quanTIseq (Finotello and Trajanoski 2018), EPIC (Racle and Gfeller 2020), CIBERSORT (Newman *et al.* 2015), TIMER (Li *et al.* 2020), xCell (Aran *et al.* 2017), and MCP-counter (Becht *et al.* 2016). In addition, we included three novel ones: ESTIMATE, ABIS, and ConsensusTME. ESTIMATE (Yoshihara *et al.* 2013) is a gene-set-based method that dissects tumor transcriptomics into its tumor, stromal, and immune components. It computes an immune and a stromal score, plus a combined “ESTIMATE” score quantifying the non-tumoral component of a sample; the latter is then used to infer tumor purity, i.e. the percentage of tumor cells in the sample. ABIS (Monaco *et al.* 2019) is a method for the deconvolution of blood samples that includes signature matrices to estimate immune-cell type fractions from RNA-seq data (17 cell types) or microarray data (11 cell types). ConsensusTME (Jiménez-Sánchez *et al.* 2019) is a method that can quantify 15 immune cell types, plus fibroblasts and endothelial, using a curated set of genes derived from an ensemble of gene-set-based methods.

Most of the methods included in immunedeconv rely on pre-built signatures or gene-sets included with their source code. Nonetheless, in their original implementation, EPIC, CIBERSORT, ConsensusTME, and seqImmuCC enabled the usage of custom gene sets or signatures provided by the user. To allow for further flexibility in immunedeconv usage, for these methods, we enabled the possibility to perform cell-type quantification based on user-provided signatures or gene sets. Users will therefore be able to use their manually-curated signatures to deconvolve bulk RNA-seq of specific tissues or organisms.

## 3 Quantification of immune, stromal, and endothelial cell types from mouse transcriptomics

We applied the extended immunedeconv to two publicly-available mouse RNA-seq datasets to showcase its value for the analysis of the murine immune-cell composition (details in Supplementary Methods). The *Chen* dataset (Chen *et al.* 2017) includes data from 12 samples from mouse immune tissues (spleen, bone marrow, blood, and lymph nodes). The *Petitprez* dataset (Petitprez *et al.* 2020) was generated from mice engrafted with the TC-1 cancer cell line, which derives from lung epithelial cells. It includes 14 samples from spleen, peritoneum, blood, and tumor. For both datasets, gold-standard cell fractions for four and twelve immune-cell types, respectively, were estimated in the original study for each sample using flow cytometry.

**Figure 2.**
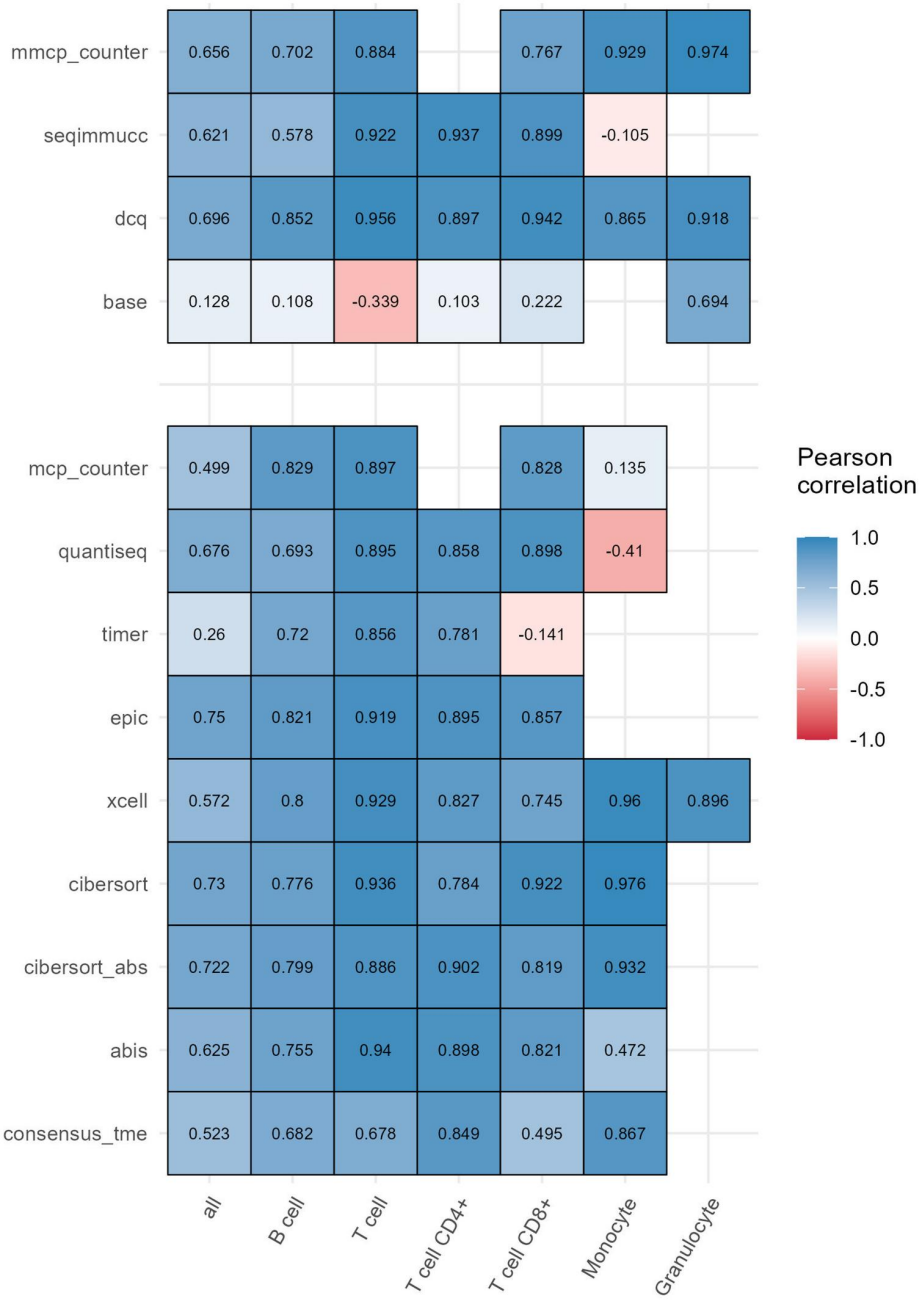
Assessment of deconvolution results for the *Chen* dataset. Pearson correlations between the cell fractions/scores estimated with different immunedeconv methods and the gold-standard cell fractions derived with flow cytometry.

We evaluated the performance of both mouse-based and human-based methods using Pearson’s correlation between the estimated and the gold-standard cell fractions derived from flow cytometry and, for the methods computing cell fractions, root-mean-square error (RMSE) (Fig. 2 and Supplementary Figs S1–S3). On both datasets, most methods obtained high, positive correlations across the various immune cells. Monocytes and T-cell subsets showed more variable results across methods in terms of correlation, while B cells showed the highest error rate (RMSE, Supplementary Figs 1–3). Overall, the performance of human-based methods was comparable to that of mouse-based ones. Although a comprehensive benchmarking is beyond the scope of the present work, these results endorse the validity of the approach based on orthologous gene mapping for the deconvolution of mouse transcriptomics.

**Figure 3.**
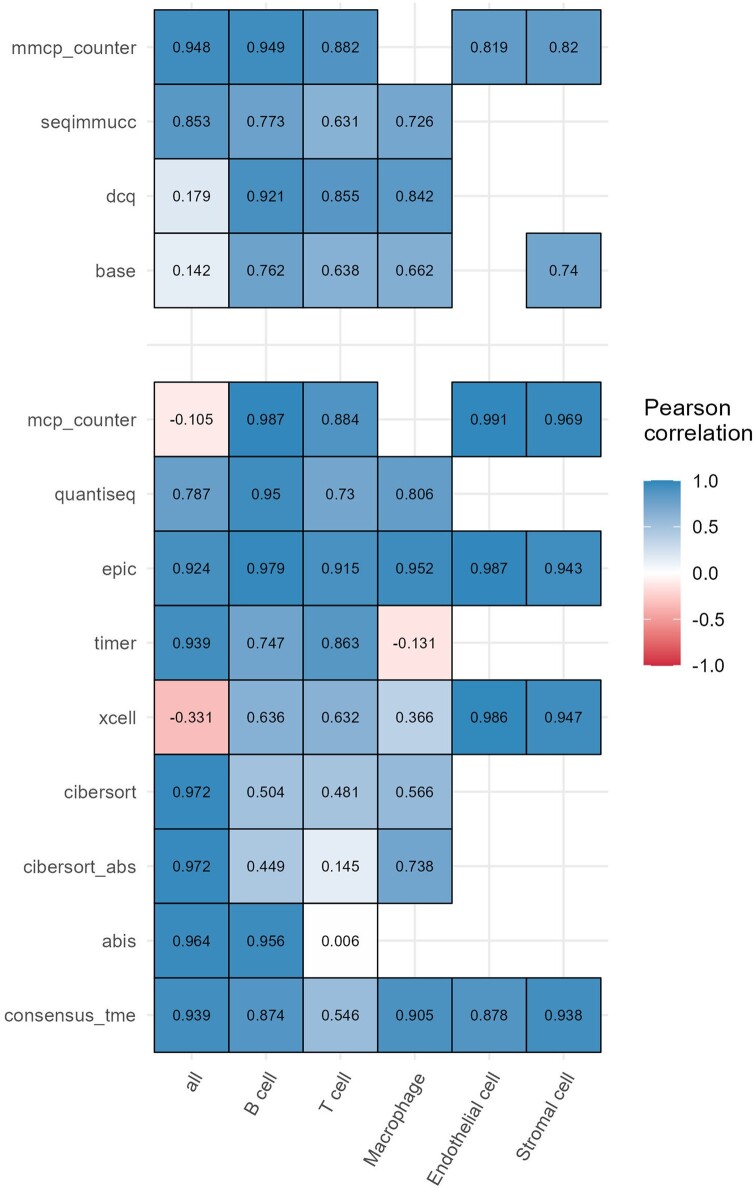
Assessment of deconvolution results for the *Tabula Muris* pseudo-bulk dataset. Pearson correlations between the cell fractions/scores estimated with different immunedeconv methods and the true cell fractions.

Due to the unavailability of RNA-seq datasets with matched gold-standard cell fractions for both immune and non-immune cells, we decided to further evaluate the methods on simulated, pseudo-bulk RNA-seq datasets. Pseudo-bulk samples can be simulated starting from single-cell RNA-seq (scRNA-seq) data, sampling cells in pre-defined proportions and summing up their expression profiles. We considered a subset of the “mammary gland” dataset from the Tabula Muris study (Tabula Muris Consortium *et al.* 2018) and simulated 20 pseudo-bulk samples using the R package SimBu (Dietrich *et al.* 2022) (details in Supplementary Methods). On this dataset, both mouse- and human-based methods were able to provide meaningful estimates not only for immune cell types, but also for endothelial and stromal cells, when predicted (Fig. 3 and Supplementary Fig. S4). As observed for the other datasets, the accuracy of T-cell quantification varied across methods, with some approaches (mMCP-counter, DCQ, EPIC, TIMER) clearly outperforming the others.

## 4 Deconvolution of mouse transcriptomics using custom gene signatures

**Figure 4.**
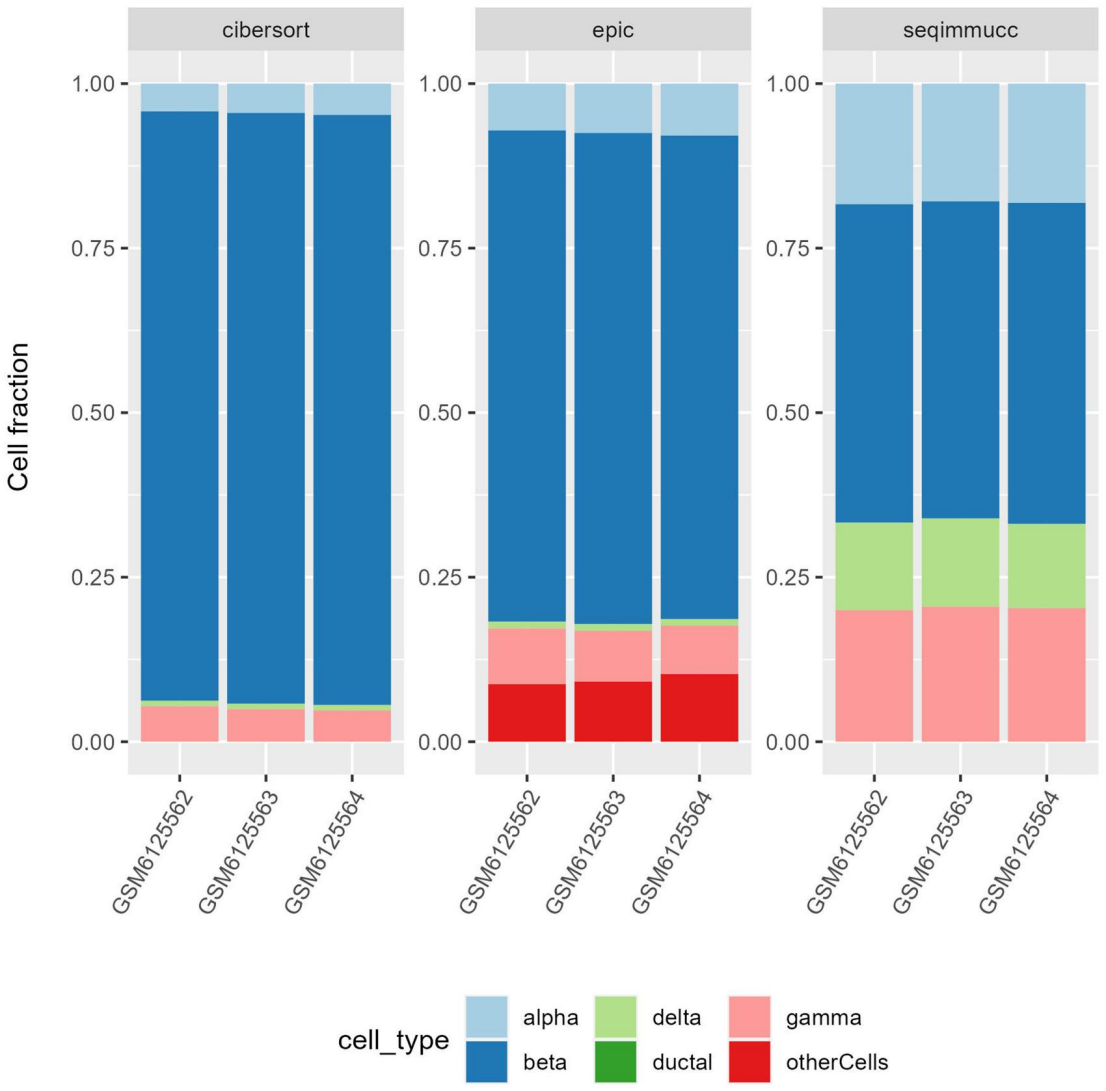
Deconvolution results for the *Tsuyama* dataset. Cell fractions obtained with CIBERSORT, EPIC, and seqImmuCC coupled with a custom signature for mouse pancreatic cell types.

To show the applicability of immunedeconv coupled with custom gene sets or signatures, we considered bulk RNA-seq from three samples from the *Tsuyama* dataset (Tsuyama *et al.* 2023), which was generated from murine pancreatic islets cultured in normoxic conditions. We derived a custom signature for five cell types (alpha, beta, gamma, delta, and ductal cells) using scRNA-seq data and cell-type-specific gene sets available from (Baron *et al.* 2016). We used these custom signatures to deconvolve the *Tsuyama* data using EPIC, CIBERSORT, and seqImmuCC, which estimate relative cell fractions (Fig. 4). All methods agreed in estimating beta cells as the most abundant cell type (75%–90% for EPIC and CIBERSORT, 50% for seqImmuCC), followed by alpha and gamma cells (4.5%–8% for EPIC and CIBERSORT, 15%–20% for seqImmuCC) in accordance with previous literature (Chen *et al.* 2022). Delta cells seem to be underestimated by most methods (<1% for EPIC and CIBERSORT, 10% for seqImmuCC), although a proper gold standard was not available to assess the methods on this specific dataset and application. Notably, by using constrained least square regression, EPIC could also estimate the percentage of unknown cellular content (10%), i.e. the fraction of cells that are present in the bulk RNA-seq data but not in the signature used for deconvolution. In this specific case, these might include endothelial, acinar, and immune cells.

## 5 Conclusions

The immunedeconv R package is a popular tool for the quantification of immune-cell types from bulk transcriptomics, but in its original implementation only supported the analysis of human data. With this extension to mouse data, we address a pressing need raised by immunedeconv users: enable the investigation of the immune-cell composition of murine tissues. Mouse models are widely used to investigate pathologies, as well as their development and evolution during treatment.

The extended immunedeconv provides methods to dissect murine transcriptomes while maintaining the same interface and ease of use. Some of the methods supported by immunedeconv offer a more global picture of the mouse immune landscape. In contrast, others allow the investigation of more specific cell types and states at high resolution. Immunedeconv allows to easily leverage their complementarities in terms of covered cell subsets and type of estimate (i.e. cell fractions vs. scores) and compare their results. These new additions, together with the possibility to use custom signatures, allow the application of immunedeconv to a wider set of tissues, cell types, and even organisms.

Of note, deconvolution methods that can learn cell-type-specific signatures from annotated scRNA-seq data have been recently introduced (Merotto et al. 2024). These methods hold great potential for the deconvolution of an increasing panel of organisms, but also present new challenges, like the difficulty of validating cell signatures derived from the input single-cell data. Unlike these approaches, immunedeconv relies on a set of well-validated methods already equipped with precomputed signatures and gene sets for human and mouse transcriptome deconvolution.

Overall, these immunedeconv extension opens new applications in the investigation of model organisms, facilitating the study of the (immune) cell composition of murine models and advancing our understanding of pathophysiology and response to treatment.

## Supplementary Material

vbae032_Supplementary_Data

## Data Availability

The Petitprez and Chen datasets were downloaded from ArrayExpress (https://www.ebi.ac.uk/biostudies/arrayexpress) using the accessions identifiers E-MTAB-9271 and E-MTAB-6458, respectively. The Tsuyama was downloaded from Gene Expression Omnibus (GEO, https://www.ncbi.nlm.nih.gov/geo) using the through the accession identifier GSE202603. No novel data was generated in this study.
