# Supplementary material for: Making mouse transcriptomics deconvolution accessible with immunedeconv
Source: Bioinform Adv. 2024 Feb 28;4(1):vbae032. doi: 10.1093/bioadv/vbae032 (PMC10924280; doi:10.1093/bioadv/vbae032)
Supplement: vbae032_Supplementary_Data [file vbae032_supplementary_data.pdf]

Supplementary Figures

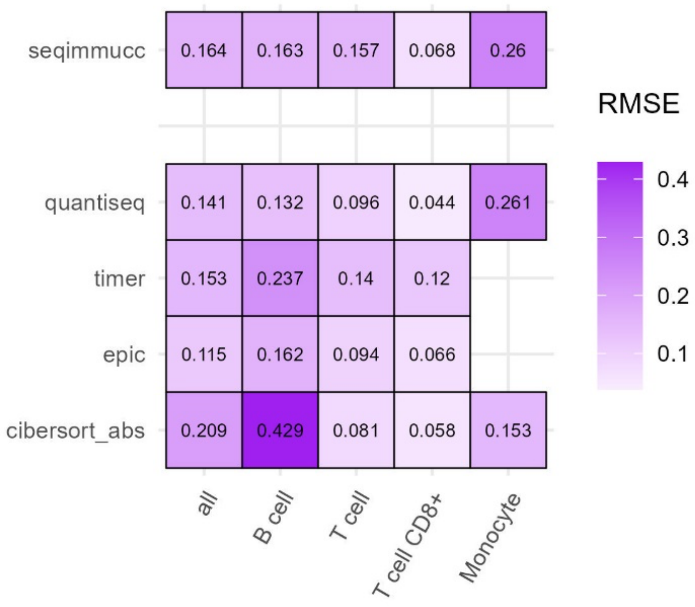

Supplementary Fig. 1: Assessment of deconvolution results for the *Chen* dataset. Root-mean-square error (RMSE) between the cell fractions estimated with different immunedeconv methods and the gold-standard cell fractions derived with flow cytometry.

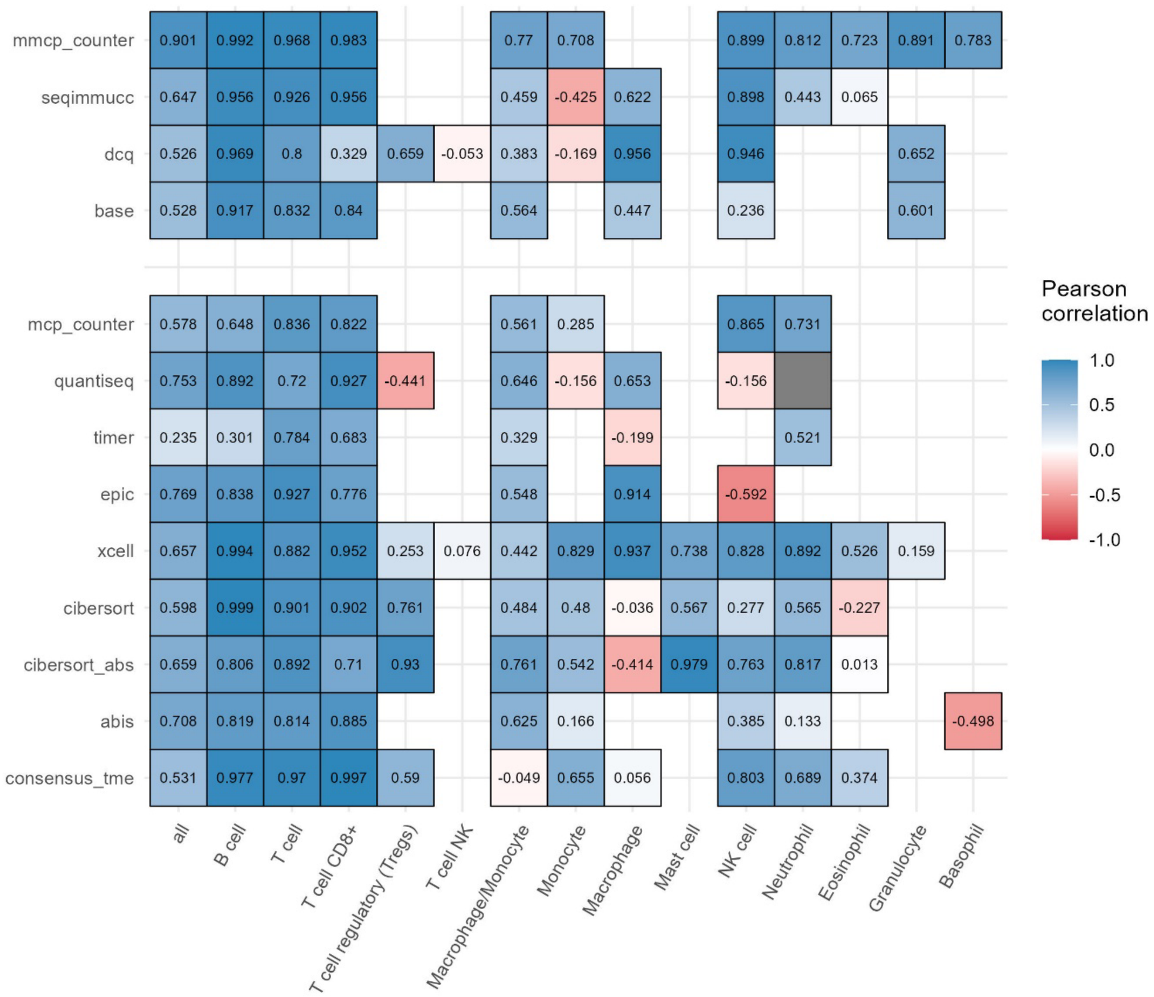

Supplementary Fig. 2: Assessment of deconvolution results for the *Petitprez* dataset. Pearson correlations between the cell fractions/scores estimated with different immunedeconv methods and the gold-standard cell fractions derived with flow cytometry. NA's, obtained for undetected cell types, are indicated in grey.

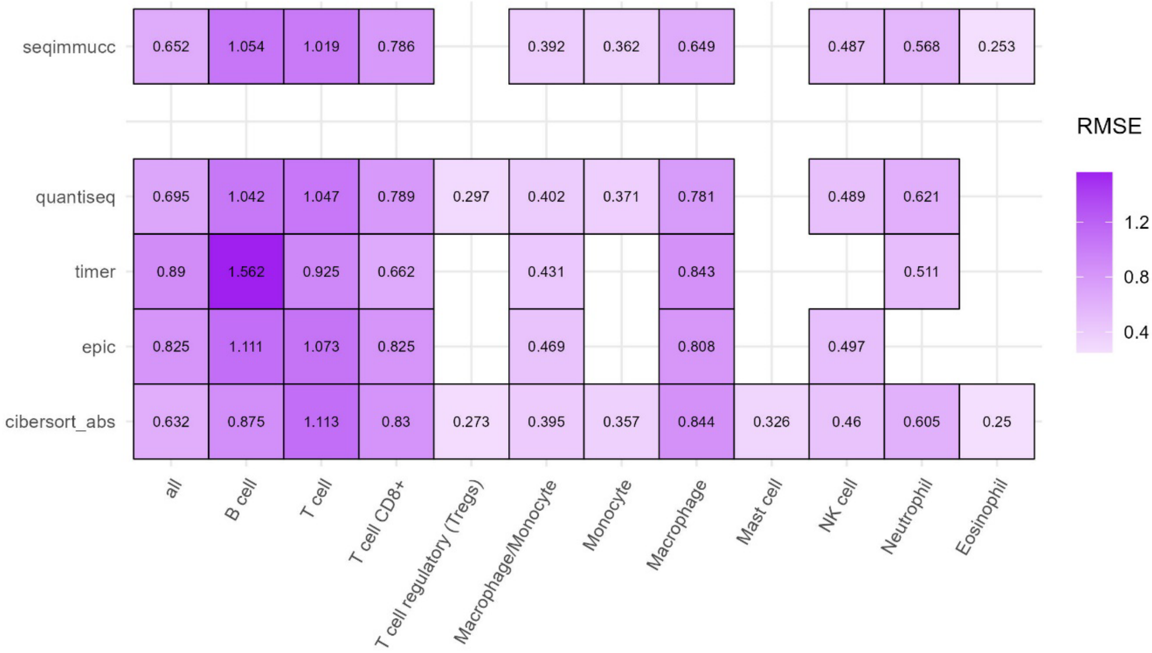

Supplementary Fig. 3: Assessment of deconvolution results for the *Petitprez* dataset. Root-mean-square error (RMSE) between the cell fractions estimated with different immunedeconv methods and the gold-standard cell fractions derived with flow cytometry.

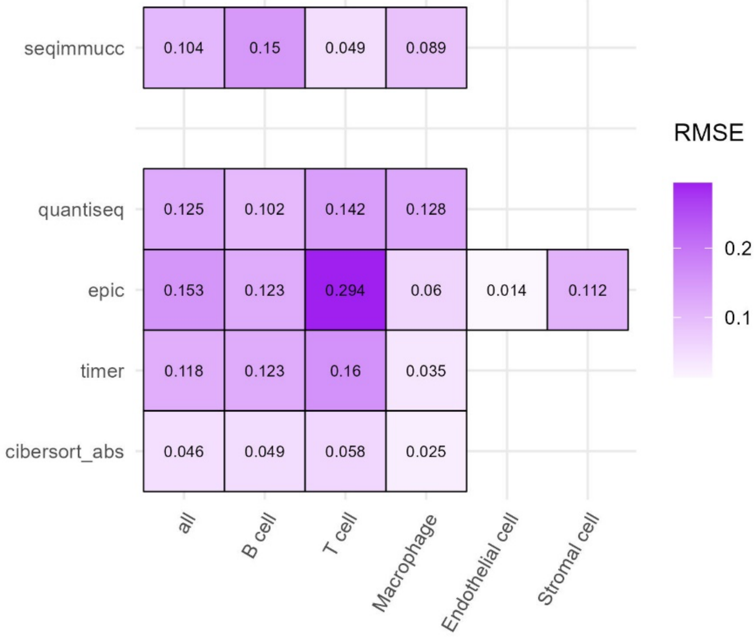

Supplementary Fig. 4: Assessment of deconvolution results for the *Tabula Muris* pseudobulk dataset. Root-mean-square error (RMSE) between the cell fractions estimated with different immunedeconv methods and the true cell fractions in the simulated pseudo-bulk data.

Supplementary Methods

Methods implementation in immunedeconv

mMCP-counter (Petitprez *et al.*) was accessed from the corresponding R package. In the original implementation of the method, the gene identifiers (Mouse Genome Informatics Gene Symbol) were based on the GRCm38 version of the murine genome. After the publication of the method, the Genome Reference Consortium (GRC) updated the reference mouse genome from the GRCm38 to the GRCm39 version. Since the alignment of raw reads to get the gene counts is usually performed on the latest version of the genome, this resulted in some marker genes missing from the bulk dataset. Therefore, we retrieved from ENSEMBL the updated Gene Symbols and updated the mMCP-counter gene signature. In turn, the mMCP-counter package was then updated to allow the choice of the version of the marker genes to use for the analysis.

The method seqImmuCC (Chen *et al.*, 2018) was originally available only as a web server. It was therefore reimplemented in immunedeconv. Deconvolution can be performed using either nu support vector regression (nu-SVR, requires the user to provide CIBERSORT R script) or linear least squares regression (LLSR). The R script for the latter was provided by the authors. The seqImmuCC RNA-seq-based signature matrix (available from https://github.com/wuaipinglab/ImmuCC) was updated with the latest Gene Symbols, as done for mMCP-counter.

The original DCQ (Altboum *et al.*, 2014) algorithm is implemented in the R ComICS package, which contains all the required files to run the analysis. It is important to note that DCQ is a method intended to be applied to differential expression data since it was first developed to find the differences in terms of cell composition across samples taken at different time points or which underwent different treatments. The authors recommend normalizing gene expressions for each sample using the mean and the standard deviation of the expression in the reference samples. Therefore, a function was implemented to perform this operation, which lets the user specify a set of samples to be considered for the normalization. If not provided, the mean expression across all samples is considered as the reference expression profile.

The BASE (Varn *et al.*, 2016) algorithm is available as an R script with the original publication. This method relies on a “cell compendium”, i.e., a signature matrix where the expression of various cell types is z-scored. We followed the authors’ instructions to build the compendium for murine hematopoietic cell types starting from several signature matrices. The resulting compendiums were tested on the Petitprez validation dataset. The signatures matrices considered were those of DCQ and seqImmuCC. In addition, the two hematopoietic mouse datasets GSE109125 and GSE15907 were accessed from the Immunological Genome Project website (https://www.immgen.org/): these contain reference transcriptomes for many hematopoietic murine cell types, as well as control samples and unassigned cells which had to be removed. The compendium obtained from GSE15907 was the one selected.

DCQ signature and BASE compendium encompass 207 and 185 fine-grained cell phenotypes, respectively, identified mainly by their surface markers. Using the cell type-specific metadata

provided by each method, we manually curated a table to assign each cell phenotype to a coarser category (e.g. from “T.4MEM.LN” to “CD4+ Memory T cell”). This table is used internally by immunedeconv once the deconvolution is performed to combine the results. The scores of the cell subtypes belonging to the same type are grouped considering either their sum in the case of DCQ, or their scaled median in the case of BASE.

Three additional human-based methods were included in the package. We implemented ABIS (Monaco *et al.*, 2019), originally available as a Shiny app, adapting its source code in the package. ConsensusTME (Jiménez-Sánchez *et al.*, 2019) was included with its own R package. ConsensusTME, like TIMER, requires the user to specify the cancer type of the samples being analyzed, allowing only one cancer type per analysis. In order to extend the analysis to a set of samples from different cancer types, we added a function to perform deconvolution multiple times, grouping samples of the same cancer type, and merging the results together. For ESTIMATE (Yoshihara *et al.*, 2013), the R package was obtained from rforge and its code and gene sets were integrated into immunedeconv.

Immunedeconv uses a cell type table to map the results of each deconvolution analysis to a controlled vocabulary to make the cell types comparable across different methodologies. The cell type table wasupdated to include all cell types estimated by the newly-introduced methods.

All human-based methods were also extended to the deconvolution of murine RNA-seq data. The principle is that the murine gene names from the bulk RNA-seq data are converted to their corresponding human orthologs, so that the human-based methods can be used for their deconvolution without changing their internal implementation and/or signature matrix/gene sets. Murine gene names are often converted to the respective human orthologs by capitalizing the letters, however this approach can be unreliable. Immunedeconv currently retrieves the human and murine genes by querying the ENSEMBL database, retrieving the MCG gene symbols and the corresponding HGNC names. In case the ENSEMBL website is unresponsive, the Mouse Genome Database is used (http://www.informatics.jax.org/downloads/reports/HOM_MouseHumanSequence.rpt).
Finally, four methods (CIBERSORT, EPIC, ConsensusTME and seqImmuCC) allow, in their native implementation, the use of a signature matrix other than the one implemented. New method-specific functions that require the necessary data to perform custom deconvolution (i.e. signature matrix, marker genes, etc.) were therefore implemented.

RNA-seq data access and processing

Raw FASTQ files were retrieved from ArrayExpress using the accession codes E-MTAB-9271 (*Petitprez dataset*) and E-MTAB-6458 (*Chen dataset*). Both datasets were processed using the nf-core/rnaseq framework (Ewels *et al.*, 2020) and the GRCm39 genome from the GRC. Briefly, FASTQ files were first aligned to the reference genome with STAR (Dobin *et al.*, 2013), and then summarized with Salmon (Patro *et al.*, 2017) to obtain both read counts and transcripts per millions (TPMs). The flow cytometry estimates for the various cell types were obtained from the authors (*Petitprez* dataset) and from the method’s Github repository (https://github.com/wuaipinglab/ImmuCC/tree/master) (*Chen* dataset), respectively.
The *Tsuyama dataset* (Tsuyama et al., 2023) was accessed from the Gene Expression Omnibus (GEO) archive through the accession number GSE202603. Along with the FASTQ files, the TPM and FPKM counts are available. The TPM values were retrieved for the samples

cultivated in normoxic conditions. The *Tabula Muris* (Tabula Muris Consortium et al., 2018) dataset, composed of single-cell RNA sequencing (scRNA-seq) data generated with the 10x Genomics technology and the associated cell-type annotations, was accessed from Figshare (https://figshare.com/projects/Tabula_Muris_Transcriptomic_characterization_of_20_organs_and_tissues_from_Mus_musculus_at_single_cell_resolution/27733).

Deconvolution of mouse RNA-seq data

All mouse deconvolution methods were run on the TPM data, according to the methods specifications. For seqImmuCC, the nu-SVR approach was used (`algorithm='SVR'`). For DCQ, the mean and standard deviation of all samples were used to scale the data (`ref.samples=NULL`). Human deconvolution methods were run on the TPM data, mapping the results to the cell types present in the flow cytometry references. For TIMER and ConsensusTME the tumor samples from the *Petitprez* were considered as lung squamous cell carcinoma (`cancer_type='LUSC'`). The remaining samples and the *Chen* dataset were considered as diffuse large B cell lymphoma (`cancer type='DLBL'`). This cancer type was determined to be the most appropriate for deconvolution using the TIMER 2.0 web app (T. Li *et al.*, 2020). For quanTIseq and EPIC, the analysis was carried separately for blood PBMC samples (`tumor=FALSE`), and on the tissue samples (`tumor=TRUE`). For methods that allow analysis of RNA-seq and microarray data (ABIS, EPIC, quanTIseq), the correction for microarray data was disabled (`arrays=FALSE`). Finally, for EPIC and quanTIseq mRNA bias correction was enabled (`scale_mrna=TRUE`).

Simulation and deconvolution of mouse mammary gland RNA-seq pseudo-bulk data

The preprocessed scRNA-seq data (gene counts) of from the Tabula Muris "mammary gland" dataset was processed with the R package Seurat (Hao et al., 2023) to extract the gene counts and the cell-type annotations. 20 pseudo-bulk samples were simulated using the R package SimBu (Dietrich et al., 2022) (simulate_bulk function) sampling 1000 cells from the dataset among B cells, T cells, macrophages, endothelial cells, and stromal cells; basal and epithelial cells were excluded from the simulation as they are not quantified by any of the immunedeconv methods. Cell fractions in the pseudo-bulk samples were based on the fractions in the single-cell dataset with an added variability (`scenario='mirror_db', balance_even_mirror_scenario=0.05`). The pseudo-bulk were simulated accounting for cell-type=specific mRNA bias (`scaling_factor='expressed_genes'`). Deconvolution was performed on the count per million (CPM)-scaled data, as for the other analyses. For ConsensusTME and TIMER, the breast cancer type was selected (`cancer_type='BRCA'`). Deconvolution was performed with the same options described above. For quanTIseq and EPIC, the option `tumor='TRUE'` was used. The stromal cells were matched with deconvolution results obtained for stromal cells (BASE, xCell) or fibroblasts (EPC, MCP and mMCP-counter, ConsensusTME).

Deconvolution of mouse pancreatic RNA-seq data using custom signatures

Four methods currently allow the use of custom signature matrices (EPIC, CIBERSORT, ConsensusTME and seqImmuCC). Since they provide cell fractions estimates, CIBERSORT, seqImmuCC and EPIC were used to deconvolve the *Tsuyama* dataset. The methods require the user to provide a signature matrix, while the latter also requires a set of marker genes for the cell types of interest, respectively. Single-cell transcriptomic profiles of the pancreatic cells

were retrieved from (Baron *et al.*, 2016) and a signature matrix was generated with CIBERSORTx (Newman *et al.*, 2019). Pancreatic Islets marker genes were accessed from the R package Bseq-SC (Baron *et al.*, 2016) (R objects `pancreasIslet` and `pancreasMarkers`). Since these were human-gene-based, we converted them to the correspondent mouse orthologous genes using the newly introduced immunedeconv function.

Supplementary Tables

Supplementary Table 1. Summary of mouse-based and human-based methods included in the extended version of immunedeconv. For each method, the name, organism, output type (scores or fractions), estimated cell types, and reference are reported.

Method	Organism	Output	Estimated cell types	Reference
mMCP-counter	Mouse	Scores	T cells, CD8+ T cells, NK cells, B cells, B cells memory, Macrophages/Monocytes, Monocytes, Granulocytes, Mast cells, Eosinophils, Neutrophils, Basophils, Vessels, Lymphatic cells, Endothelial cells, Fibroblasts	(Petitprez *et al.*)
seqImmuCC	Mouse	Immune cell fractions	CD4+ T cells, CD8+ T cells, Dendritic cells, Eosinophils, Macrophages, Mast cells, Monocytes, Neutrophils, NK cells	(Chen *et al.*, 2018)
DCQ	Mouse	Scores	B cells, Naive B cells, Myeloid/Plasmacytoid dendritic cells, Granulocytes, Macrophages, Stem cells, Monocytes, NK cells, NK T cells, Immature T cells, Naive CD4+/CD8+ T cells, Regulatory T cells, Memory CD4+ T cells, Memory/Effector memory CD8+ T cells, Gamma Delta T cells, Stromal cells	(Altboum *et al.*, 2014)
BASE	Mouse	Scores	B cells, Naive B cells, Myeloid/Plasmacytoid dendritic cells, Granulocytes, Macrophages, Stem cells, Monocytes, NK cells, Immature T cells, Naive CD4+/CD8+ T cells, Memory CD4+ T cells, Memory/Effector memory CD8+ T cells, Gamma Delta T cells, Stromal cells	(Varn *et al.*, 2016)
quanTIseq	Human	Absolute cell fractions	B cells, monocytes, classically activated (M1) macrophages, alternatively activated (M2) macrophages, neutrophils, natural killer cells, CD4+ T cells, CD8+ T cells, CD4+ regulatory T cells, dendritic cells, other uncharacterized cells	(Finotello and Trajanoski, 2018)
EPIC	Human	Absolute cell fractions	B cells, CD4+ T cells, CD8+ T cells,macrophages, natural killer cells, endothelial cells, cancer-associated fibroblasts, other uncharacterized cells.	(Racle and Gfeller, 2020)
CIBERSORT	Human	Immune/absolute cell fractions	Naïve CD4+ T cells, resting memory CD4+ T cells, activated memory CD4+ T cells, follicular helper T cells, gamma-delta T cells, CD4+ regulatory T cells, CD8+ T cells, resting natural killer cells, activated natural killer cells, naïve B cells, memory B cells, plasma cells, monocytes, unstimulated (M0) macrophages, classically activated (M1) macrophages, alternatively activated (M2) macrophages, resting mast cells, activated mast cells, resting dendritic cells, activated dendritic cells, neutrophils, eosinophils	(Newman *et al.*, 2015)
TIMER	Human	Absolute cell fractions	Dendritic cells, macrophages, neutrophils, CD8+ T cells, CD4+ T cells, B cells.	(B. Li *et al.*, 2020)
MCP-counter	Human	Scores	T cells, CD8+ T cells, cytotoxic lymphocytes, natural killer cells, B cells, cells from monocytic lineage, myeloid dendritic cells, neutrophils, endothelial cells, fibroblasts	(Becht *et al.*, 2016)
xCell	Human	Scores	Activated dendritic cells, adipocytes,	(Aran *et al.*,

			alternatively activated macrophages (M2), astrocytes, B cells, basophils, classically activated macrophages (M1), CD4+ central memory T cells, CD4+ effector memory T cells, CD4+ memory T cells, CD4+ naïve T cells, CD4+ T cells, CD8+ central memory T cells, CD8+ effector memory T cells, CD8+ naïve T cells, CD8+ T cells, chondrocytes, class-switched memory B cells, common lymphoid progenitors, common myeloid progenitors, conventional dendritic cells, dendritic cells, endothelial cells, eosinophils, epithelial cells, erythrocytes, fibroblasts, gamma-delta T cells, granulocytes, macrophage progenitors, hematopoietic stem cells, hepatocytes, immature dendritic cells, keratinocytes, lymphatic endothelial cells, macrophages, mast cells, megakaryocyte–erythroid progenitors, megakaryocytes, melanocytes, memory B cells, mesangial cells, mesenchymal stem cells, microvascular endothelial cells, monocytes, multipotent progenitors, myocytes, naïve B cells, natural killer cells, natural killer T cells, neurons, neutrophils, osteoblasts, pericytes, plasma cells, plasmacytoid dendritic cells, platelets, preadipocytes, pro B cells, regulatory T cells, sebocytes, skeletal muscle cells, smooth muscle cells, type 1 helper T cells, type 2 helper T cells	2017)
ABIS	Human	Scores	Naive CD4+ T cells, Memory CD4+ T cells, naive CD8+ T cells, Memory CD8+ T cells, MAIT T cells, VD2/Non-VD2 Gamma Delta T cells, Memory B cells, Naive B cells, Immature plasma B cells, Neutrophils, Basophils, Myeloid/Plasmacytoid dendritic cells, NK cells, Conventional/Non conventional monocytes	(Monaco *et al.*, 2019)
Consensus TME	Human	Scores	CD4+ T cells, CD8+ T cells, Gamma Delta T cells, Regulatory T cells, B cells Classically/Alternatively activated macrophages, Endothelial cells, Fibroblasts, Monocytes, Cytotoxic cells, Myeloid dendritic cells, Eosinophils, Macrophages, Mast cells, NK cells	(Jiménez-Sánchez *et al.*, 2019)
ESTIMATE	Human	Scores	Immune and Stromal scores, Estimate score, tumor purity fraction	(Yoshihara et al., 2013)

References

Altboum,Z. *et al.* (2014) Digital cell quantification identifies global immune cell dynamics during influenza infection. *Mol. Syst. Biol.*, **10**, 720.

Aran,D. *et al.* (2017) xCell: digitally portraying the tissue cellular heterogeneity landscape. *Genome Biol.*, **18**, 220.

Baron,M. *et al.* (2016) A Single-Cell Transcriptomic Map of the Human and Mouse Pancreas Reveals Inter- and Intra-cell Population Structure. *Cell Syst*, **3**, 346–360.e4.

Becht,E. *et al.* (2016) Estimating the population abundance of tissue-infiltrating immune and stromal cell populations using gene expression. *Genome Biol.*, **17**, 218.

Chen,Z. *et al.* (2018) seq-ImmuCC: Cell-Centric View of Tissue Transcriptome Measuring Cellular Compositions of Immune Microenvironment From Mouse RNA-Seq Data. *Front. Immunol.*, **9**, 1286.

Dietrich,A. et al. (2022) SimBu: bias-aware simulation of bulk RNA-seq data with variable cell-type composition. Bioinformatics, 38, ii141–ii147.

Dobin,A. *et al.* (2013) STAR: ultrafast universal RNA-seq aligner. *Bioinformatics*, **29**, 15–21.

Ewels,P.A. *et al.* (2020) The nf-core framework for community-curated bioinformatics pipelines. *Nat. Biotechnol.*, **38**, 276–278.

Finotello,F. and Trajanoski,Z. (2018) Quantifying tumor-infiltrating immune cells from transcriptomics data. *Cancer Immunol. Immunother.*, **67**, 1031–1040.

Hao,Y. *et al.* (2023) Dictionary learning for integrative, multimodal and scalable single-cell analysis. *Nat. Biotechnol.*

Jiménez-Sánchez,A. *et al.* (2019) Comprehensive Benchmarking and Integration of Tumor Microenvironment Cell Estimation Methods. *Cancer Res.*, **79**, 6238–6246.

Li,B. *et al.* (2020) Computational Deconvolution of Tumor-Infiltrating Immune Components with Bulk Tumor Gene Expression Data. *Methods Mol. Biol.*, **2120**, 249–262.

Li,T. *et al.* (2020) TIMER2.0 for analysis of tumor-infiltrating immune cells. *Nucleic Acids Res.*, **48**, W509–W514.

Monaco,G. *et al.* (2019) RNA-Seq Signatures Normalized by mRNA Abundance Allow Absolute Deconvolution of Human Immune Cell Types. *Cell Rep.*, **26**, 1627–1640.e7.

Newman,A.M. *et al.* (2019) Determining cell type abundance and expression from bulk tissues with digital cytometry. *Nat. Biotechnol.*, **37**, 773–782.

Newman,A.M. *et al.* (2015) Robust enumeration of cell subsets from tissue expression profiles. *Nat. Methods*, **12**, 453–457.

Patro,R. *et al.* (2017) Salmon provides fast and bias-aware quantification of transcript expression. *Nat. Methods*, **14**, 417–419.

Petitprez,F. *et al.* The murine Microenvironment Cell Population counter method to estimate abundance of tissue-infiltrating immune and stromal cell populations in murine samples using gene expression.

Racle,J. and Gfeller,D. (2020) EPIC: A Tool to Estimate the Proportions of Different Cell Types from Bulk Gene Expression Data. *Methods Mol. Biol.*, **2120**, 233–248.

Tabula Muris Consortium et al. (2018) Single-cell transcriptomics of 20 mouse organs creates a Tabula Muris. Nature, 562, 367–372.

Tsuyama,T. et al. (2023) Hypoxia causes pancreatic β-cell dysfunction and impairs insulin secretion by activating the transcriptional repressor BHLHE40. EMBO Rep., 24, e56227.

Varn,F.S. *et al.* (2016) Integrative analysis of breast cancer reveals prognostic haematopoietic activity and patient-specific immune response profiles. *Nat. Commun.*, **7**, 10248.

Yoshihara,K. *et al.* (2013) Inferring tumour purity and stromal and immune cell admixture from expression data. *Nature Communications*, **4**.
